# REDD1 attenuates cholestatic liver fibrosis and suppresses PI3K/AKT/mTOR pathway

**DOI:** 10.3389/fmed.2025.1628260

**Published:** 2025-09-24

**Authors:** Xiaonan Li, Xin Liu, Xinrui Shi, Zixu Li, Qizhi Shuai, Tingjuan Huang, Yun Liu, Junjie Ren

**Affiliations:** ^1^Department of Radiotherapy, Shanxi Provincial People's Hospital Affiliated to Shanxi Medical University, Taiyuan, China; ^2^Department of Gastroenterology, The First Hospital of Shanxi Medical University, Taiyuan, China; ^3^Department of Thyroid Surgery, The First Hospital of Shanxi Medical University, Taiyuan, China

**Keywords:** primary biliary cholangitis, bile duct ligation, liver fibrosis, REDD1, PI3K/AKT/mTOR

## Abstract

**Introduction:**

Liver fibrosis is reversible. Cholestasis is an important factor causing liver fibrosis. However, there are currently no effective anti-fibrotic drugs for cholestatic liver fibrosis in clinical practice.

**Methods:**

mRNA sequencing was performed using mouse bile duct ligation (BDL) of liver tissue, and RT-qPCR was used to screen for the target gene REDD1. Immunohistochemistry was used to detect the expression of REDD1, CD68, α-SMA, and PI3K/AKT/mTOR signaling pathways in primary biliary cholangitis (PBC) patient liver tissue. Subsequently, adenovirus mediated REDD1 was transfected into mouse liver tissue via tail vein to evaluate its therapeutic effect.

**Results:**

RNA sequencing revealed REDD1 was significantly upregulated in BDL-induced fibrotic liver tissue. REDD1 expression correlated positively with α-SMA and CD68 in PBC patients, suggesting its involvement in fibrogenesis. However, REDD1 overexpression ameliorated BDL-induced liver injury, reduced serum ALT/AST levels, and decreased collagen deposition, as evidenced by histological and molecular analyses (α-SMA and collagen I), indicating that REDD1 exhibited compensatory elevation in liver fibrosis. Additionally, PI3K/AKT/mTOR pathway was involved in the improvement of liver fibrosis by REDD1.

**Conclusions:**

These findings highlight REDD1 as a potential therapeutic target for liver fibrosis, acting probably through modulation of the PI3K/AKT/mTOR pathway to mitigate fibrotic processes.

## 1 Introduction

Cholestasis, whose total incidence in patients with chronic liver disease is about 10.26% (1). Cholestatic liver diseases including primary biliary cholangitis (PBC), an autoimmune disease of the liver characterized by progressive cholestasis have a higher incidence in hepatopathy (2, 3), which lead to progressive loss of liver function, liver fibrosis and subsequent liver cirrhosis, even hepatocellular carcinoma (HCC) (4, 5). Due to the reversibility of liver fibrosis, how to suppress or even reverse hepatic fibrosis has become a hot spot in the current study (6). Therefore, it is highly desirable to identify new therapeutic targets to treat liver fibrosis.

It is widely recognized that activated hepatic stellate cells (HSCs) play a pivotal role in the development of liver fibrosis (7). Several studies have shown that in both parenchymal and cholestatic liver injury, activated HSCs are the main source of extracellular matrix (ECM), including fibril-forming type I collagens (8), which disturbing normal hepatic function and architecture (9). Thus, the inhibition of HSCs activation is proposed as a considerable way to alleviate liver fibrosis (10). Ursodeoxycholic acid (UDCA), a hydrophilic bile acid, has been widely used clinically as a safe medical therapy for liver diseases, especially for the PBC and cholestatic liver disease (11–13). Although UDCA can decrease cholesterol content, stimulate biliary secretion, alleviate clinical symptoms and improve disease outcomes, it cannot exert anti-fibrotic effects by reducing HSCs activation (14–16). Up till now, there are no clinically effective drugs to inhibit HSCs activation and reduce liver fibrosis (17).

REDD1 (Regulated in development and DNA damage response 1), which is also known as Ddit 4 (DNA damage inducible transcript 4), is a nutrient/energy sensor and an early stress-response gene activated by hypoxia, depletion of growth factors, DNA damage as well as glucocorticoids (1–3). REDD1 is a gene activated by stress that regulates a range of cellular activities, such as metabolism, oxidative stress, autophagy, and cell destiny, playing a role in the development of metabolic and inflammatory disorders, neurodegenerative conditions, and cancer (4). There are currently multiple theories regarding the role of REDD1 in treating liver diseases. A study shown that lack of REDD1 prevented non-alcoholic fatty liver disease (5, 6). By contrary, overexpression of REDD1 attenuated HSCs activation and CCL4-induced liver fibrosis via inhibiting of TGFβ/Smad signaling pathway (7). However, the role of REDD1 in cholestatic liver fibrosis is currently unclear.

Here, we employed RNA sequencing data analysis to screen for REDD1 in mice cholestatic liver fibrosis models. More importantly, liver tissues from PBC patients were used to verify it. Given the previous references and experiments, we hypothesized that REDD1 had a protective effect on cholestatic liver fibrosis, therefore REDD1 was overexpressed in BDL mice to validate its therapeutic effect, aiming to provide a potential therapeutic target for clinical anti-liver fibrosis treatment.

## 2 Materials and methods

### 2.1 Establishment of mice common bile duct ligation and Ad-REDD1 treatment

The animal study was reviewed and approved by the Animal Ethics Committee of Shanxi Medical University, and the license Key was SCXY2023-0006. Male C57BL/6 mice were purchased from Animal Center of Shanxi Medical University and housed in pathogen-free facilities. The animal experiment was approved by the Institutional Animal Care and Use Committee of The First Hospital of Shanxi Medical University. Cholestatic liver fibrosis was induced by ligation of the common bile duct for 4 weeks as described previously (8), and liver tissues were obtained 4 weeks after operation.

Adenovirus-mediated REDD1 (Ad-REDD1) was synthesized by Shanghai Sangon Biotechnology Co., Ltd. Two weeks after BDL, adenovirus expressing REDD1 was systemically administered via tail vein injection (once a week for a total of 2 weeks). Serum and liver tissue were collected at the end of 4 weeks.

### 2.2 Patient samples

Ten normal liver tissues were obtained from healthy liver transplant donors undergoing hepatic resection and regarded as controls in this study. Twenty PBC liver tissues were obtained from patients with PBC confirmed by pathological tests at Beijing Friendship hospital. All study procedures followed the ethical standards and were approved by the Ethics Committee for Medicine of Beijing Friendship Hospital, Capital Medical University (number: 2020-P2-118-01).

### 2.3 Library building and RNA sequencing

Three samples in each group (control group: C1, C2, C3; experimental group: B1, B2, B3) were randomly selected. Liver tissues were taken out according to the manufacturer's protocol and sent to Gene Denovo Biotechnology Co, Ltd (Guangzhou, China) for RNA extraction, RNA quality inspection, library preparation and quality inspection, and finally for RNA sequencing and analysis. To ensure sequencing quality, we use strict quality control to ensure the construction quality of the library. The testing standards are as follows: (1) Agarose gel electrophoresis: analyze the integrity of sample RNA and whether there is DNA contamination; (2) NanoPhotometer spectrophotometer: detects RNA purity (OD260/280 and OD260/230 ratio); (3) Qubit2.0 Fluorometer: accurate quantification of RNA concentration; (4) Agilent 2100 Bioanalyzer: accurately detects RNA integrity. The steps for filtering reads are as follows: (1) Remove reads containing adapters; (2) Remove reads with N ≥ 10%; (3) Remove all reads that are A bases; (4) Remove low-quality reads (more than 50% of the total reads are alkaline with a quality value Q ≤ 20). illumina sequencing platform was used.

### 2.4 Differentially expressed genes (DEGs) analysis and functional enrichment

Differential expression analysis was performed using the DESeq R package. We screened genes with FDR < 0.05 and | log2FC | > 1 as significantly different genes. GO enrichment analysis and KEGG pathway analysis of DEGS were employed by the GOseq R packages and KOBAS software. A hypergeometric *p* < 0.05 was considered to be significantly enriched.

### 2.5 H&E staining

Liver tissues were fixed and paraffin-embedded, followed by sliced. H&E staining were performed using Hematoxylin and Eosin (HE) staining kit (G1120, Solarbio, Beijing, China) according to standard protocols.

### 2.6 Sirius red staining

The liver tissue sections were deparaffinized and hydrated, followed by staining with Sirius red solution (G1472, Solarbio, Beijing, China) for 30–60 min (adjusted based on collagen content). After rinsing off excess dye, the sections were dehydrated through a series of ethanol concentrations and cleared in xylene, finally mounted with neutral resin and observed under a microscope.

### 2.7 Masson staining

The liver tissue sections were performed by Masson stain kit (MSC-8003, MXB, China). It primarily used dyes such as hematoxylin, eosin, and aniline blue to selectively stain different tissue components, with collagen fibers appearing blue and muscle fibers appearing red under the microscope.

### 2.8 ALT and AST detection

Alanine Aminotransferase (ALT, SEA207Mu, Cloud-Clone Corp Wuhan) and Aspartate Aminotransferase (AST, SEB241Mu, Cloud-Clone Corp Wuhan) were determined in mouse serum by ELISA.

### 2.9 Verification of gene using quantitative real-time polymerase chain reaction (qRT-PCR)

mRNA was extracted from liver tissues using TRIzol solution (Takara, shiga, Japan) per the manufacturer's instructions, and followed by the concentration and purity of mRNA were measured. cDNA Reverse Transcription Kit (QIAGEN) and miScript SYBR Green PCR Kit (QIAGEN) were used for reverse transcription and quantitative real-time PCR of mRNA. GAPDH was identified to calculate the expression levels of mRNA. The required primers were designed and synthesized by Sangon Biotech (Shanghai, China), and the gene sequences were provided in Table 1.

**Table 1:** Primers sequences for qRT-PCR.

**Gene**	**Forward primer (5^′^3^′^)**	**Reverse primer (5^′^3^′^)**
sgkl	CGTCCGAACGGGACAACAT	GTCCACCGTCCGGTCATAC
REDDI	TAAGTTCTGCCAACTCTTCCTT	CGGAGCTGTAGAGTTTCTTCTT
Ngf	CCAGTGAAATTAGGCTCCCTG	CCTTGGCAAAACCTTTATTGGG
Colla2	TCGTGCCTAGCAACATGCC	TTTGTCAGAATACTGAGCAGCAA
Collal	TAAGGGTCCCCAATGGTGAGA	GGGTCCCTCGACTCCTACAT
Igha	TGTGGGTGAACTGGATTCTACT	ACCGACCCTTAACAGATGCAG
Spp1	CACTCCAATCGTCCCTACAGT	CTGGAAACTCCTAGACTTTGACC
GAPDH	CCACTCACGGCAAATTCAAC	CTCCACGACATACTCAGCAC

### 2.10 Immunohistochemical (IHC)

Paraffin-embedded liver tissue sections were dewaxed with xylene and then hydrated with gradient ethanol. After antigen repairing, the slices were incubated with primary antibodies at 4 °C overnight as follow: anti-REDD1 (ab106356, Abcam, 1:200), anti-CD68 (ab283654, Abcam, 1:200), anti-α-SMA (ab7817, Abcam, 1:200), anti-p-PI3K (T40065, abmart, 1:400), anti-p-AKT (T40067, abmart, 1:100), anti-mTOR (T56571, abmart, 1:100). Then the samples were subjected to goat anti-rabbit HRP-conjugated secondary antibody (ab6721, Abcam, 1:10000) at room temperature for 2 h. Finally, the slices were stained with DAB, counterstained, dehydrated and sealed. The positive judgment criteria were as following: the cytoplasm or nucleus was stained with brown granules. The subsequent quantitative analysis was blinded.

### 2.11 Statistical analysis

SPSS 23.0 statistical software and GraphPad Prism 8.0 were used to analyze calculations. All datas were represented as the mean ± standard deviation (SD). For comparison between two groups, *t*-test is used for normal distribution, and Mann Whitney U-test or Wilcoxon rank test is used for non-normally distribution; Comparing between three or more groups, one way ANOVA followed by Tukey test was employed for analyzing the normally distributed data, while Kruskal-Wallis test followed by Dunn's *post-hoc* test was employed for analyzing the non-normally distributed data. Pearson or Spearman correlation coefficients were calculated to assess relationships between biomarkers and REDD1. This test was selected due to the linear relationship between biomarkers and REDD1. These biomarkers were included based on their biological relevance to the study hypotheses. Prior to data analysis, no normalization was required as variables exhibited approximately normal distributions. *p*-*value* < 0.05 showed a statistically significant difference.

## 3 Results

### 3.1 mRNA sequencing of liver tissue with fibrosis induced by BDL

**Figure 1:**
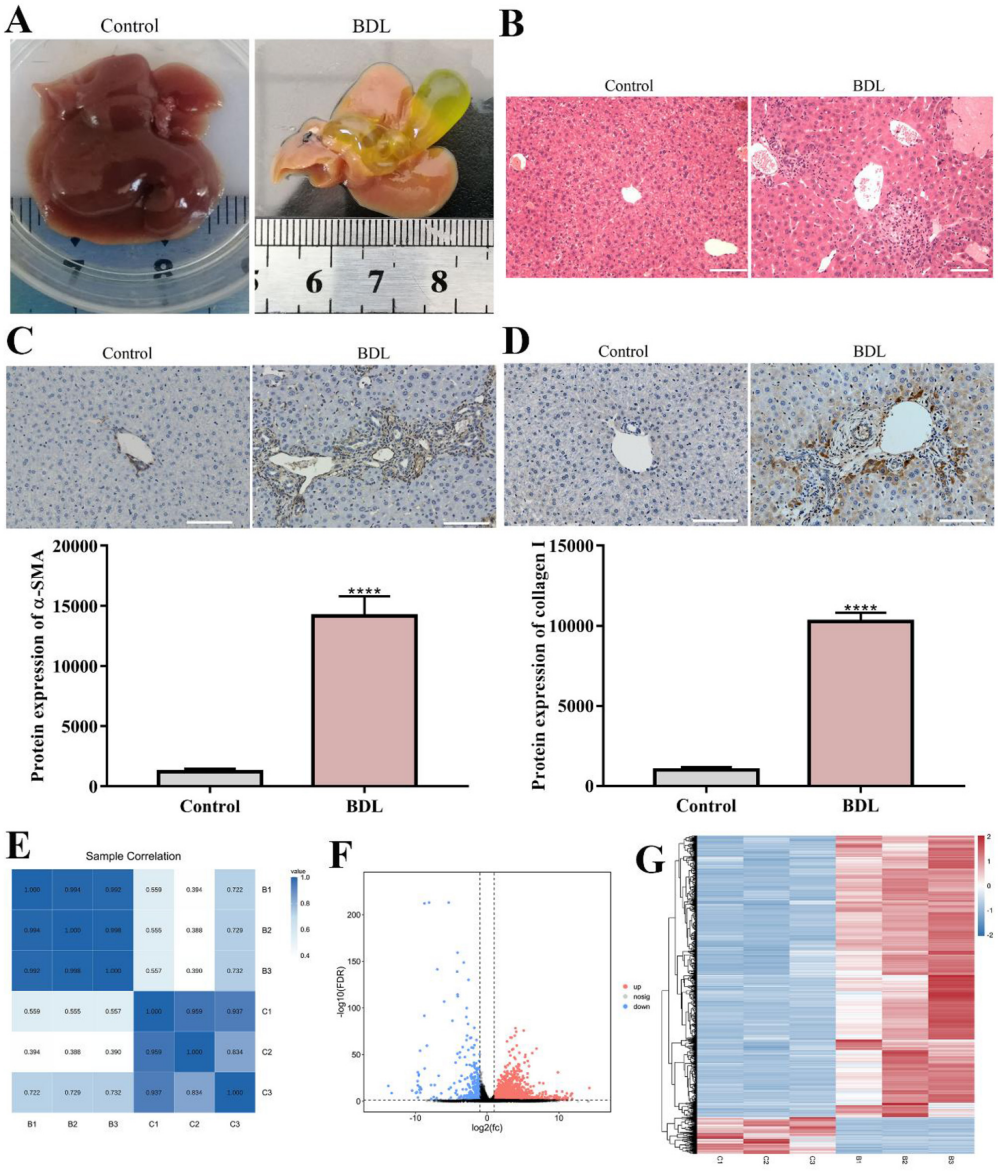
mRNA sequencing of liver tissue with fibrosis induced by BDL. **(A)** General view of liver (*n* = 3). **(B)** H&E was performed to evaluate the establishment of hepatic fibrosis model (*n* = 3). Scale bar: 100 μm. **(C, D)** Immunohistochemistry analysis of α-SMA and collagen I in control and BDL group (*n* = 3). Scale bar: 100 μm. **(E)** Correlation coefficient plot. Right and bottom of plot represent sample names, and left and top of plot represent sample clustering. The higer the correlation coefficient, the stronger the correlation between two samples. **(F)** Volcano map. X-axis indicates log fold change of differential expression genes between BDL and control groups. And y-axis indicates –log 10 of adjusted *p-value*. Each point represents a gene. Red points present significantly upregulated genes, blue points present significantly downregulated genes, and gray points were genes with not significantly different expression, which met screening condition of FC > 1, adjusted *p*-value (adj *P*) < 0.05. **(G)** Differential genes cluster heat map. It was a hierarchical clustering analysis of up- and down-regulated genes. Red and blue, respectively, indicate up- and down-regulated genes. ****P < 0.0001. All data are presented as the mean ± SD.

To verify the successful establishment of cholestatic hepatic fibrosis model, we carried out general view of liver and H&E staining. As shown in Figure 1A, compared with control group, the color of liver tissue in BDL was yellow, the texture became hard while the edge became blunt. At the same time, the gallbladder silted bile, gradually increased. H&E staining of the liver tissues from BDL showed the lesions were mainly concentrated around the interlobular bile ducts, manifested as infiltration of inflammatory cells, increase in centrilobular necrosis, proliferation and dilation of small bile ducts, enlargement of lumen, and formation of collagen fibers (Figure 1B). Similarly, Immunohistochemistry analysis of fibrosis markers, including α-SMA and collagen I, were performed to assess the degree of liver fibrosis in BDL mouse. As expected, the expression of α-SMA (*p* < 0.0001) and collagen I (*p* < 0.0001) were significantly increased in BDL group compared to control (Figures 1C, D). All these results confirmed the model of liver fibrosis was established successfully.

To further investigate the molecular mechanism of cholestatic hepatic fibrosis, we performed RNA sequencing to detect the difference of transcriptome with or without BDL in triplicate. According to the comparison results of total mapped reads that can be located on the genome, we calculated the distribution position of reads in the reference genome, more than 88% of the clean reads can be compared to the exon region (Supplementary Figure S1A). After obtaining the FPKM value, we showed the difference of gene expression distribution between control and BDL group through the expression distribution map (Supplementary Figures S1B, C). To identify DEGs with adjusted *p* < 0.05, DESeq R package was adopted. A total of 3,224 DEGs were obtained from control and BDL group, among which, 2,852 genes were upregulated in BDL group as well as 372 genes were downregulated (Figures 1E–G).

### 3.2 Screening and validation of REDD1 in BDL-induced liver tissue

**Figure 2:**
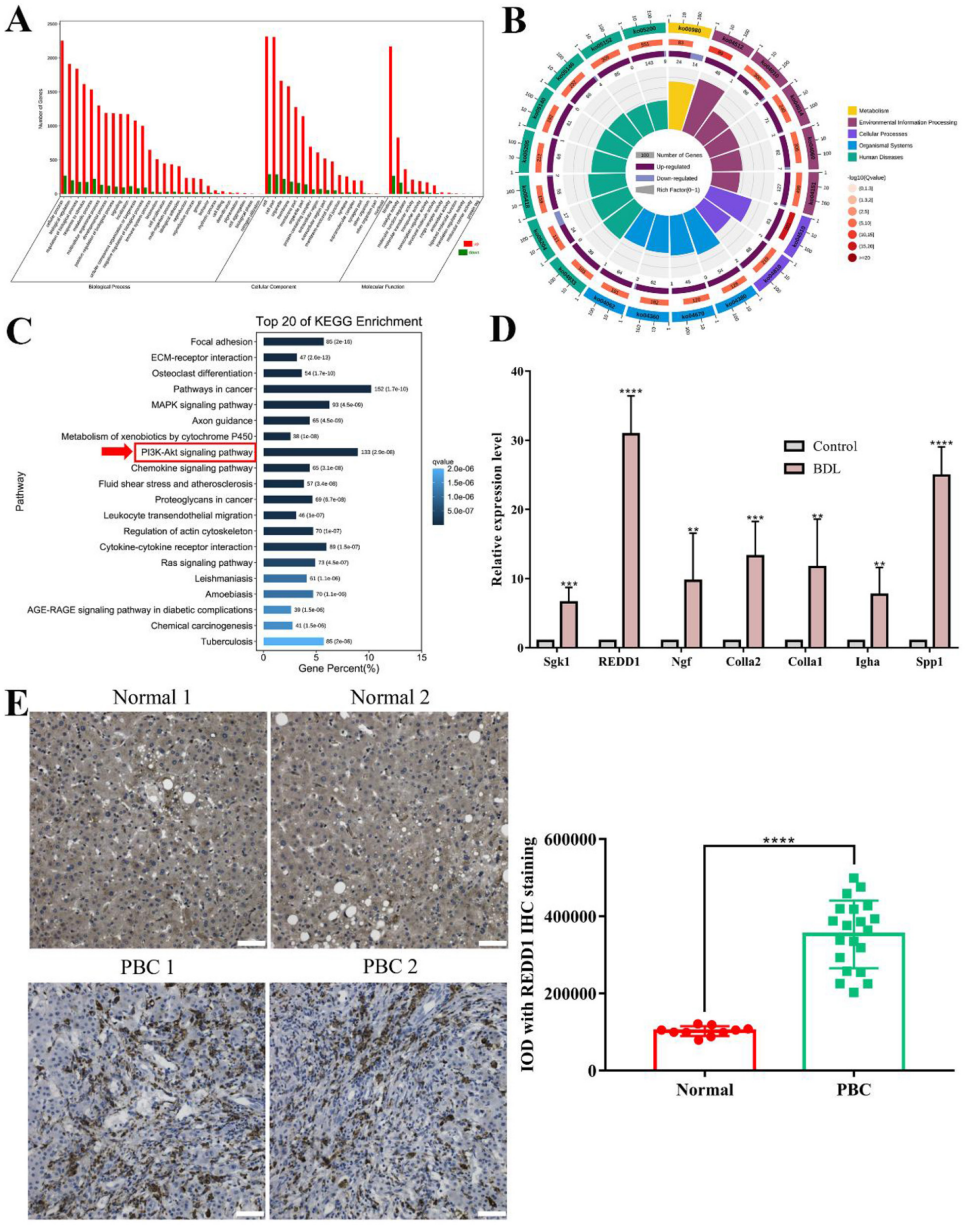
Screening and validation of REDD1 in BDL-induced liver tissue. **(A)** GO classification of DEGs. X-axis represents different GO subcategories, y-axis represents the number of DEGs in each GO subcategory. **(B)** KEGG enrichment circle diagram. The first circle represents the coordinate ruler of the number of DEGs. Different colors represent different A class. The second circle represents the number and q-value of pathway. The third circle represents the up-regulated and down-regulated DEGs in this pathway. The fourth circle represents all DEGs in this pathway. **(C)** KEGG classification of DEGs. X-axis represents gene percent to each pathway. The darker the color, the smaller the q-value. Y-axis represents different pathways. **(D)** Relative expression level of seven DEGs by RT-qPCR in control and BDL group (*n* = 3). **(E)** Immunohistochemistry analysis of REDD1 in normal (*n* = 10) and PBC patient (*n* = 20) liver tissues. Scale bar: 50 μm. **P < 0.01, ***P < 0.001, ****P < 0.0001. All data are presented as the mean ± SD.

To illustrate the functional changes of gene expression of BDL-induced hepatic fibrosis, a GO enrichment analysis of 3,224 DEGs from three libraries was carried out. We summed up a total of 58 enriched GO terms (*p* < 0.05), 28 categories in biological processes, 17 categories in cellular component, 13 categories in molecular function. In biological process category, the significant enrichment included cellular process, biological regulation, regulation of biological process, response to stimulus, metabolic process. Among these, cellular process is the most enriched with 2,253 genes up-regulated and 270 genes down-regulated (Figure 2A). To further investigate the possible signaling pathways that DEGs may participate in, KEGG pathway enrichment analysis was conducted. A total of 340 pathways were annotated and the top 20 pathways were mainly concentrated in five categories, including metabolism, environmental information processing, cellular processes, organismal systems, human diseases (Figure 2B). KEGG analyzed a variety of molecular pathways, such as signal transduction-associated pathways, including MAPK signaling pathway (ko04010), PI3K/AKT signaling pathway (ko04151), Ras signaling pathway (ko04014), TNF signaling pathway (ko04668), TGF-beta signaling pathway (ko04350). Infectious diseases-associated pathways, including Leishmaniasis (ko05140), Amoebiasis (ko05146), Tuberculosis (ko05152). Cancers-associated pathways, including pathways in cancer (ko05200), proteoglycans in cancer (ko05205), chemical carcinogenesis (ko05204). Figure 2C showed that in the top 20 pathways, both up-regulated and down-regulated genes were enriched in pathways in cancer and PI3K/AKT signaling pathway.

Under adjusted *P* < 0.05 and *log2FC* >1 criteria, we got 2,852 upregulated DEGs. Our previous studies found that autophagy was implicated in the development and progression of hepatic fibrosis (9), we further screened based on the autophagy pathway PI3K/AKT, and 127 DEGs were identified in total. Next, based on the comprehensive evaluation of *P-value* and the expression difference multiple, 7 DEGs including SGK1 (serum and glucocorticoid regulated kinase 1), REDD1, NGF (Nerve Growth Factor), Col1α2, Col1α1, IGHA (immunoglobulin heavy alpha chain), and Spp1 (Secreted phosphoprotein 1) were identified. To validate the expression of 7 DEGs in RNA-seq data, we performed RT-qPCR analysis and observed most significantly upregulation of REDD1 (*p* < 0.0001) in BDL group (Figure 2D). To understand the role of REDD1 in human PBC, we examined the expression of REDD1 in liver tissue sections of patients diagnosed with PBC. As shown in Figure 2E, there were almost no positive cells in normal liver tissue, the brownish staining in the image of normal liver tissue was non-specific staining, while REDD1 positive cells appeared in PBC liver tissue (*p* < 0.0001), which was brownish and mainly concentrated in the bile duct.

### 3.3 REDD1 is correlated with liver fibrosis

**Figure 3:**
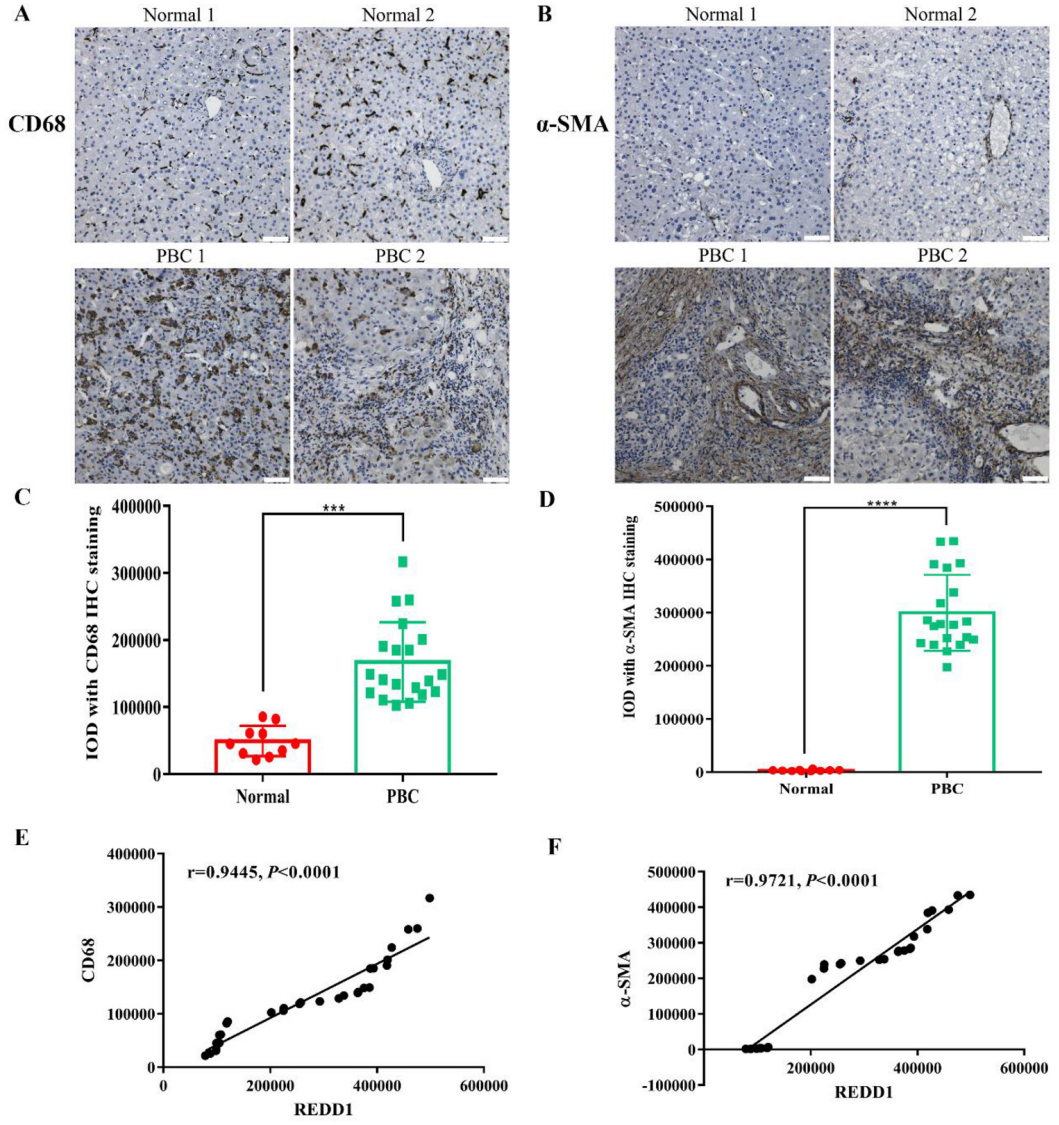
REDD1 is correlated with liver fibrosis. **(A, B)** Immunohistochemistry analysis of CD68 and α-SMA in normal (*n* = 10) and PBC patient (*n* = 20) liver tissues. Scale bar: 50 μm. **(C, D)** Statistical significance was determined by one-way ANOVA with Dunnett's multiple comparisons test. **(E, F)** Correlation between the expression of REDD1 and CD68, α-SMA. ***P < 0.001, ****P < 0.0001. All data are presented as the mean ± SD.

To determine whether REDD1 is associated with fibrosis in PBC patients, we immunohistochemically stained PBC liver sections for CD68 and α-SMA. CD68 is commonly considered to be a pan-macrophage marker (10), which can reflect the severity of liver fibrosis. The results showed that the expression of CD68 was low in control group (*p* < 0.001), but high in PBC patients (Figures 3A, C). In addition, the expression of α-SMA was significantly increased (*p* < 0.0001) in patients with PBC (Figures 3B, D). To explore the expression correlation between REDD1 and CD68 (*r* = 0.9445, *p* < 0.0001) or α-SMA (*r* = 0.9721, *p* < 0.0001), correlation analysis was performed. The results showed that both of them were positively correlated with REDD1 (Figures 3E, F), which suggested that REDD1 might modulate the development of fibrosis in patients with PBC.

### 3.4 REDD1 may be associated with PI3K/AKT/mTOR pathway in liver fibrosis

**Figure 4:**
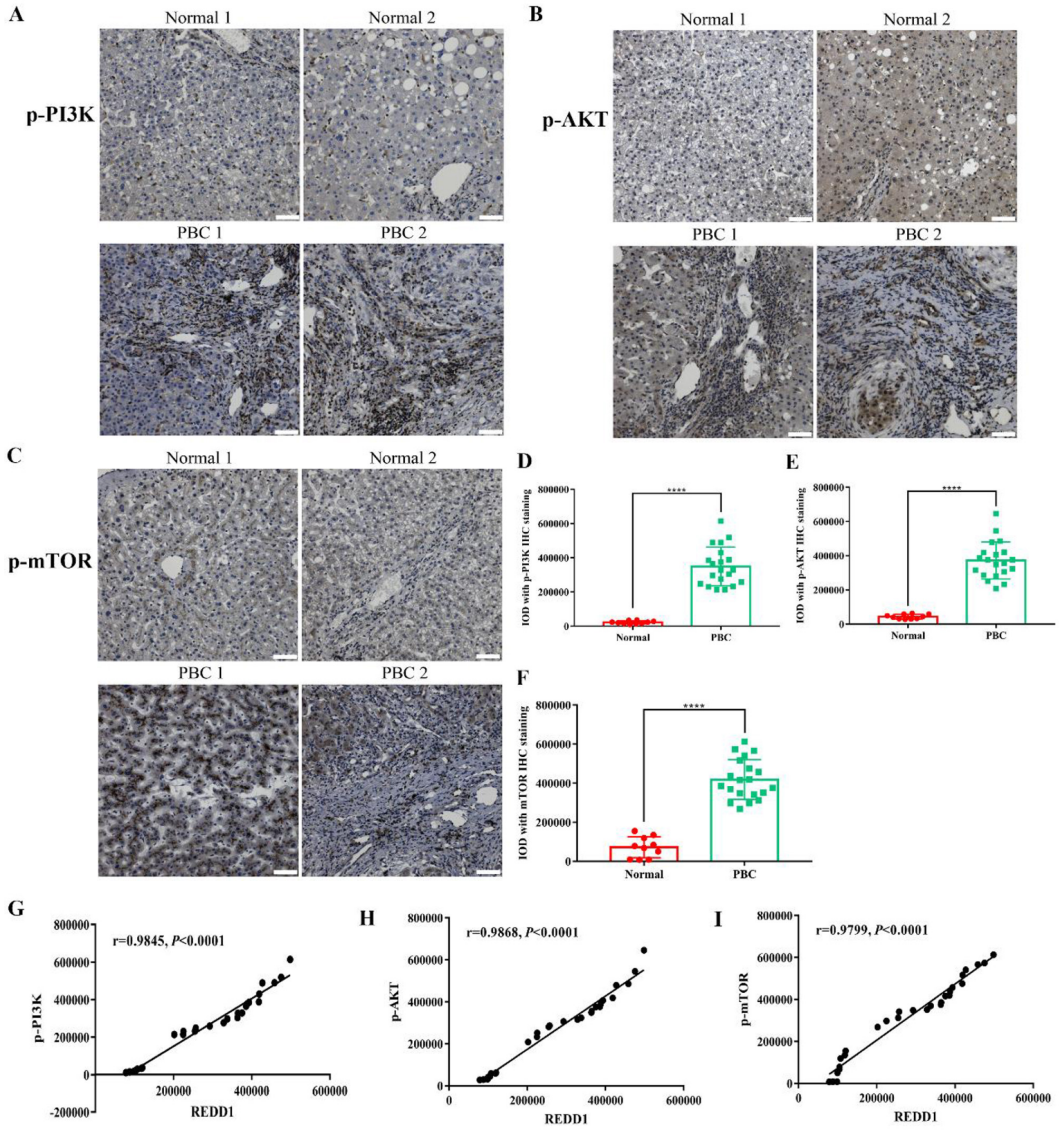
REDD1 may be associated with PI3K/AKT/mTOR pathway in liver fibrosis. **(A–C)** Immunohistochemistry analysis of p-PI3K, p-AKT, and p-mTOR in normal and PBC patient liver tissues. Scale bar: 50 μm. **(D–F)** Statistical significance was determined by one-way ANOVA with Dunnett's multiple comparisons test. **(G–I)** Correlation between the expression of REDD1 and p-PI3K, p-AKT, p-mTOR. ****P < 0.0001. All data are presented as the mean ± SD.

Increasing researches indicated that activation of the PI3K/AKT/mTOR signaling pathway promoted activation of HSCs and hepatic fibrosis (11, 12). To validate the involvement of PI3K/AKT/mTOR in PBC patients, PI3K/AKT/mTOR activity was determined. The results showed that the protein expression of p-PI3K (*p* < 0.0001), p-AKT (*p* < 0.0001), p-mTOR (*p* < 0.0001) increased in PBC patients compared to normal group (Figures 4A–F), suggesting that PI3K/AKT/mTOR might be activated in clinical cholestatic liver fibrosis. To further study whether REDD1 was associated with PI3K/AKT/mTOR pathway, we conducted a correlation analysis. As expected, REDD1 had a significant correlation with p-PI3K (*r* = 0.9845, *p* < 0.0001), p-AKT (*r* = 0.9868, *p* < 0.0001), p-mTOR (*r* = 0.9799, *p* < 0.0001) (Figures 4G–I), demonstrating REDD1 might be involved in the progression of liver fibrosis through PI3K/AKT/mTOR pathway.

### 3.5 REDD1 ameliorates BDL-induced liver injury and reduces hepatic fibrosis indices

**Figure 5:**
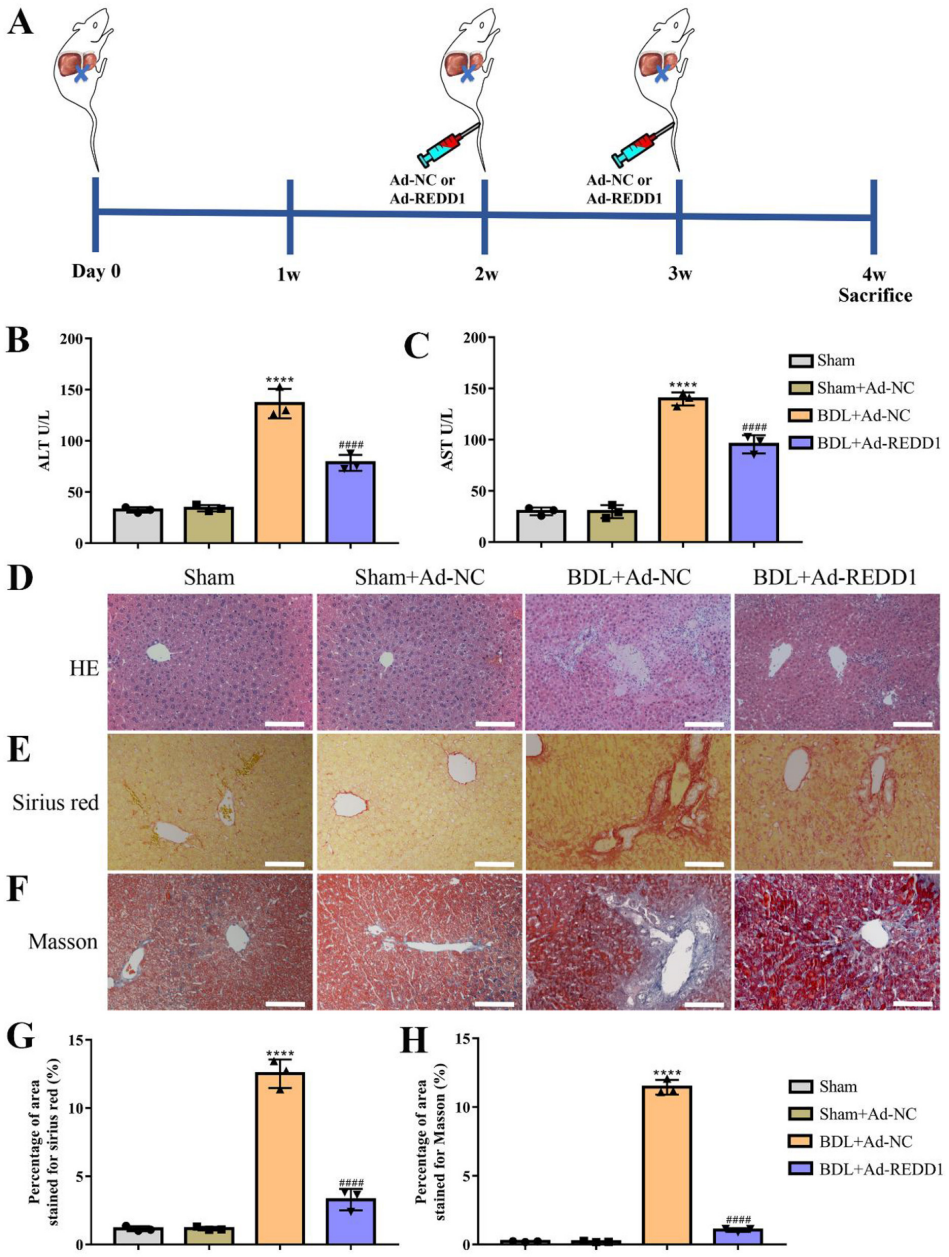
REDD1 ameliorates BDL-induced liver injury and reduces hepatic fibrosis indices. **(A)** Scheme of the establishment of liver fibrosis mouse model induced by BDL and schedule of treatments by Ad-REDD1. **(B, C)** ALT and AST concentrations in the serum of control mice and the mice with liver fibrosis induced by BDL after Ad-REDD1 treatment (*n* = 3). **(D, E, F)** Representative images of H&E staining, Sirius Red staining and Masson staining in control mice and the mice with liver fibrosis induced by BDL after Ad-REDD1 treatment (*n* = 3), demonstrating the degree of liver inflammation and fibrosis. Scale bars: 100 μm. **(G, H)** Quantitative analysis of Sirius Red staining and Masson staining in control mice and the mice with liver fibrosis induced by BDL after Ad-REDD1 treatment (*n* = 3). ****P < 0.0001, ^####^*P* < 0.0001. All data are presented as the mean ± SD.

To further assess the therapeutic potential of REDD1, the mice models of liver fibrosis induced by BDL were treated with adenoviral mediated REDD1 (Ad-REDD1) (Figure 5A). Compared to untreated controls, REDD1 significantly decreased serum ALT (*p* < 0.0001) and AST (*p* < 0.0001) levels in BDL-induced fibrotic mice, indicating amelioration of liver injury (Figures 5B, C). Representative images of H&E staining, Sirius Red staining, and Masson staining revealed a reduced infiltration of inflammatory cells, weakened bile duct dilation, and decreased collagen fibers in Ad-REDD1-treated mice compared to untreated controls (Figures 5D–F). Quantitative analysis further confirmed that Ad-REDD1 treatment significantly reduced collagen deposition, as evidenced by decreased Sirius Red (*p* < 0.0001) and Masson staining (*p* < 0.0001) intensities (Figures 5G, H). In conclusion, REDD1 effectively mitigated BDL-induced liver fibrosis by reducing liver injury and suppressing collagen accumulation.

### 3.6 REDD1 inhibits hepatic fibrogenic gene/protein expression

**Figure 6:**
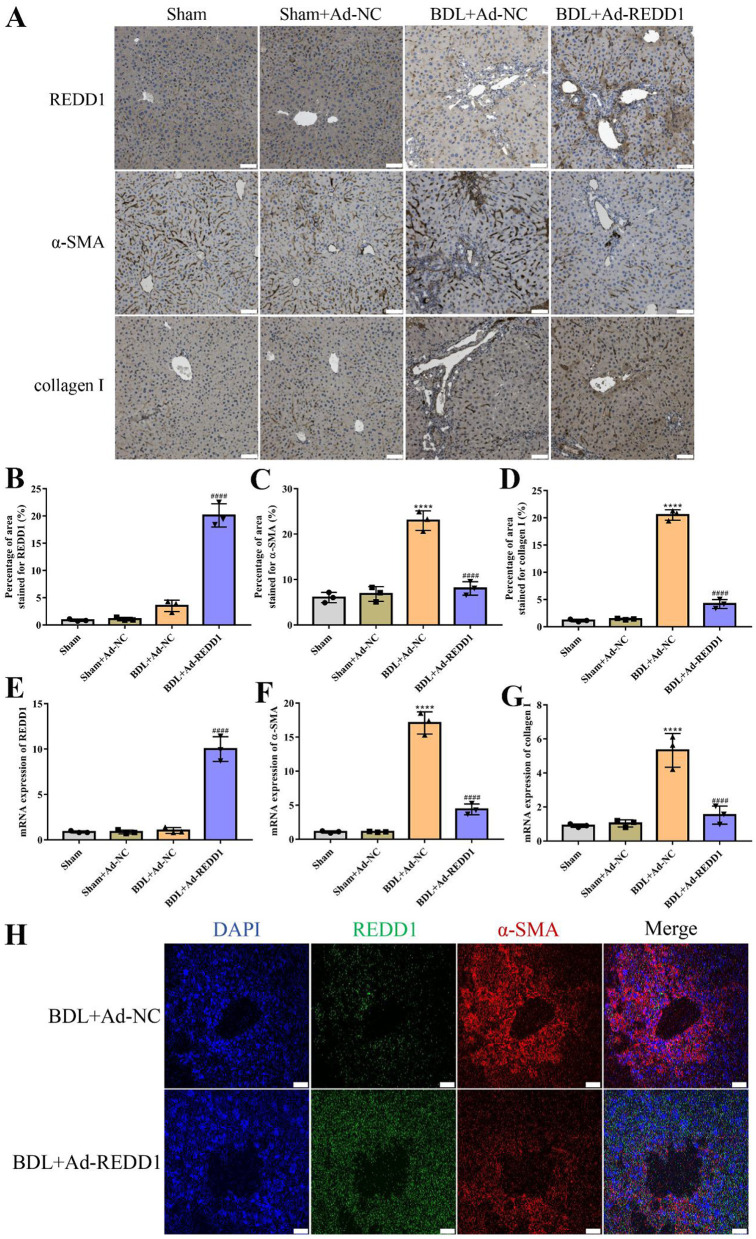
REDD1 inhibits hepatic fibrogenic gene/protein expression. **(A)** Immunohistochemistry analysis of REDD1, α-SMA and collagen I in control mice and the mice with liver fibrosis induced by BDL after Ad-REDD1 treatment (*n* = 3). Scale bar: 50 μm. **(B–D)** Quantitative analysis of REDD1, α-SMA and collagen I in control mice and the mice with liver fibrosis induced by BDL after Ad-REDD1 treatment (*n* = 3). **(E–G)** RT-qPCR analysis of REDD1, α-SMA and collagen I mRNA levels in control mice and the mice with liver fibrosis induced by BDL after Ad-REDD1 treatment (*n* = 3). **(H)** Co-immunofluorescence staining of REDD1 (red) and α-SMA (green) in BDL mice with or without Ad-REDD1 treatment (*n* = 3). DAPI was used to visualize nuclei (blue). Scale bars: 200 μm. ****P < 0.0001, ^####^*P* < 0.0001. All data are presented as the mean ± SD.

To further evaluate the anti-fibrotic effects of REDD1, fibrogenic markers such as α-SMA and collagen I, which are indicators of hepatic stellate cell activation, were examined. Compared to controls, REDD1 (*p* < 0.0001) was obviously increased in the adenovirus-transfected experimental group (Figures 6A, B, E), Indicating REDD1 was successfully overexpression. Immunohistochemistry analysis showed a significant reduction in the expression of α-SMA (*p* < 0.0001) and collagen I (*p* < 0.0001) in Ad-REDD1-treated mice compared to untreated BDL-induced fibrotic mice (Figure 6A), which was corroborated by quantitative analysis indicating decreased protein levels of these fibrogenic markers (Figures 6C, D). Additionally, RT-qPCR analysis revealed that Ad-REDD1 treatment significantly downregulated the mRNA levels of α-SMA (*p* < 0.0001) and collagen I (*p* < 0.0001) in the liver tissues of BDL-induced fibrotic mice (Figures 6F, G). Besides, co-immunofluorescence staining of REDD1 and α-SMA revealed that REDD1 overexpression increased the α-SMA puncta area, implying that REDD1 might exert its anti-hepatic fibrosis effect by acting on HSCs (Figure 6H). These findings suggested that REDD1 ameliorated liver fibrosis by suppressing the expression of key fibrogenic genes and proteins, further supporting its role in mitigating BDL-induced liver injury and fibrosis.

### 3.7 PI3K/AKT/mTOR pathway is involved in the therapeutic effect of REDD1 on liver fibrosis

**Figure 7:**
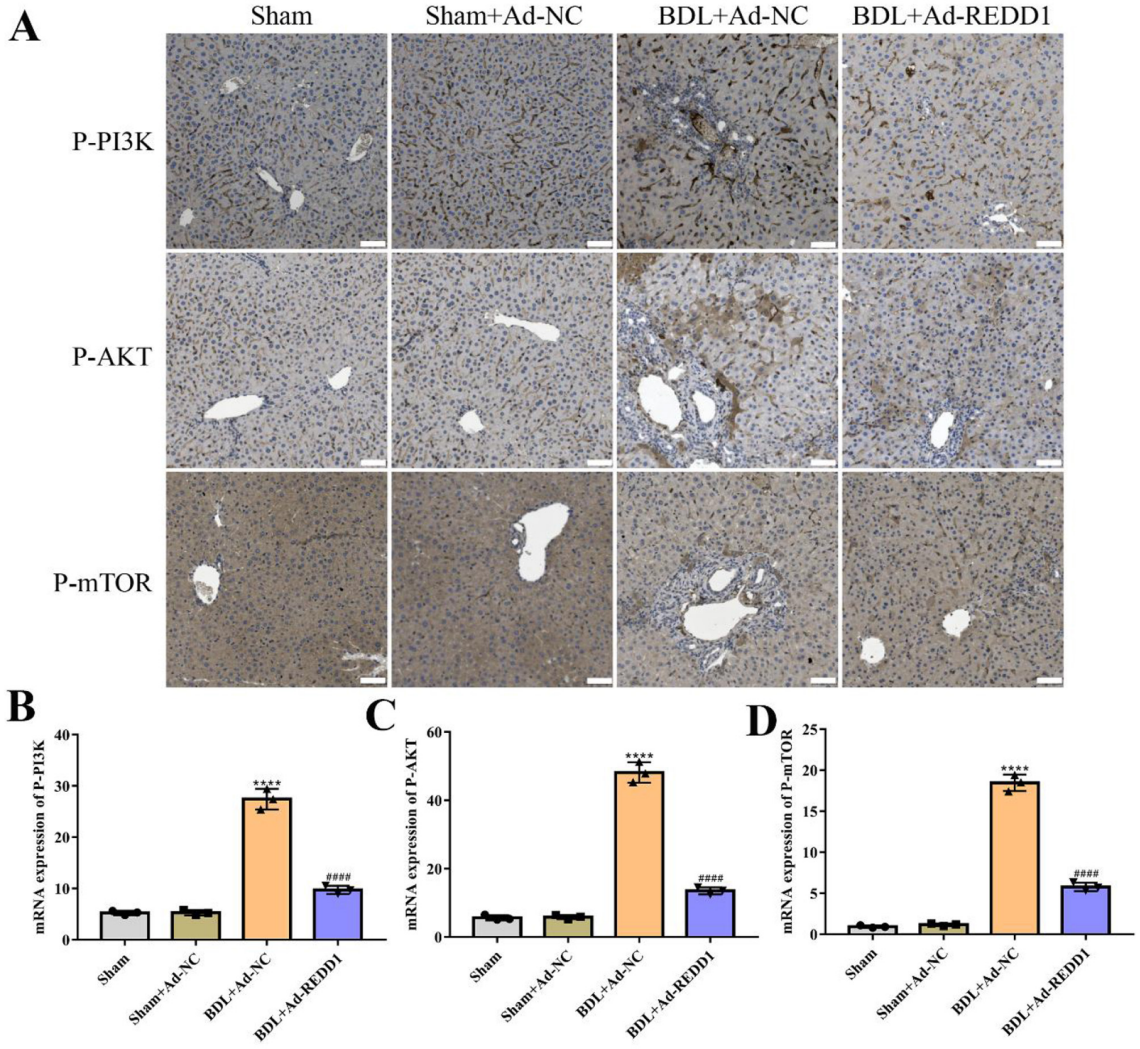
PI3K/AKT/mTOR pathway is involved in the therapeutic effect of REDD1 on liver fibrosis. **(A)** Immunohistochemistry analysis of P-PI3K, P-AKT, and P-mTOR in control mice and the mice with liver fibrosis induced by BDL after Ad-REDD1 treatment (*n* = 3). Scale bar: 50 μm. **(B–D)** Quantitative analysis of P-PI3K, P-AKT, and P-mTOR in control mice and the mice with liver fibrosis induced by BDL after Ad-REDD1 treatment (*n* = 3). ****P < 0.0001, ^####^*P* < 0.0001. All data are presented as the mean ± SD.

PI3K/AKT/mTOR signaling pathway plays a critical role in the development and progression of liver fibrosis. Inhibition of PI3K/AKT/mTOR pathway has been shown to suppress HSC activation and reduce fibrotic markers (13, 14). In this study, immunohistochemistry analysis revealed a significant reduction in the expression of phosphorylated PI3K (P-PI3K), AKT (P-AKT), and mTOR (P-mTOR) in Ad-REDD1-treated mice compared to untreated BDL-induced fibrotic mice (Figure 7A). Quantitative analysis further confirmed that Ad-REDD1 treatment markedly decreased the levels of P-PI3K (*p* < 0.0001), P-AKT (*p* < 0.0001), and P-mTOR (*p* < 0.0001) (Figures 7B–D), suggesting that REDD1 exerts its anti-fibrotic effects by inhibiting the activation of the PI3K/AKT/mTOR signaling pathway. These findings highlighted the involvement of PI3K/AKT/mTOR pathway in mediating the protective role of REDD1 against BDL-induced liver fibrosis.

## 4 Discussion

PBC is a chronic intrahepatic cholestatic hepatopathy. Due to the lack of effective treatment so far, it can eventually develop from early hepatic fibrosis into end-stage liver disease with the need for liver transplantation. Common bile duct ligation in mice is a classic method to produce an animal model of cholestatic liver fibrosis (15). There are increasing evidences that alterations in transcriptome are associated with human disease, ranging from cancer to autoimmune diseases to cardiovascular pathology (16–18). Herein we performed RNA high-throughput sequencing to explore the variations of transcriptome of hepatic fibrosis induced by BDL.

Consistent with our previous study (9), the appearance of many pathological features including the enlarged gallbladder, bile duct dilatation, and collagen fiber formation indicated that BDL-induced hepatic fibrosis model was established successfully, which makes preparations for the next step of sequencing. Through researching differential gene expression, we looked forward to discover probable gene targets of BDL-induced hepatic fibrosis, and further described underlying signaling pathways, which may provide a new idea for the clinical treatment of cholestatic liver fibrosis.

Through transcriptome information analysis, a total of 239,727,428 clean reads were obtained. After data acquisition and comparative analysis, we obtained the FPKM values of 22,299 genes for subsequent analysis, and finally got 3,224 DEGs. The current study showed a significantly altered of mRNA in BDL-induced liver fibrosis. Previously, we found that autophagy promoted HSCs activation, thus exacerbating liver fibrosis (9). To obtain candidate response genes related to autophagy, we screened DEGs based on *p*-*values*, log2FC and autophagic pathway PI3K/AKT/mTOR, and ultimately got 7 DEGs, including SGK1, REDD1, NGF, Col1α2, Col1α1, IGHA, Spp1.

REDD1 exhibits protective functions by regulating multiple intrinsic cell activities through mammalian target of rapamycin complex 1 (mTOR1)-dependent or -independent mechanism (4). Increasing evidences suggest that REDD1 regulates various cellular and metabolic processes, including autophagy (4, 19, 20). Through the validated assays, REDD1 was found to be elevated in the liver tissue of PBC patients, but surprisingly, REDD1 overexpression ameliorated BDL-induced liver function and liver fibrosis. The reason might be a feedback regulation mechanism that the upregulation of REDD1 may be a compensatory increase, representing a compensatory mechanism to counteract fibrotic progression and hepatocyte injury, reflecting an adaptive response to chronic cholestatic injury. REDD1 had a potential protective in cholestatic liver diseases, and could be a target gene for cholestatic hepatic fibrosis. However, due to the inherent lack of cellular specificity in adenovirus vectors, REDD1 was overexpressed in all hepatic cell types after Ad-REDD1 administration, it is currently unknown which specific cells in the liver are targeted by REDD1 to exert its anti-liver fibrosis effects. Further research is required to identify these target cells.

The PI3K/AKT/mTOR signaling pathway plays a pivotal role in the development and progression of liver fibrosis by regulating key cellular processes such as HSCs activation, proliferation, and survival. Activation of this pathway promotes the transformation of quiescent HSCs into myofibroblasts, which are responsible for ECM deposition, a hallmark of fibrosis. It was reported that inhibition of the PI3K/AKT/mTOR pathway attenuated liver fibrosis (21). Therefore, the activation of PI3K/AKT/mTOR pathway found in PBC patients might be strongly associated with the occurrence of cholestatic liver fibrosis in clinical. The involvement of the PI3K/AKT/mTOR pathway in mediating the anti-fibrotic effects of REDD1 highlights its role in modulating key signaling cascades implicated in liver fibrosis. However, further research is needed to elucidate the regulation for REDD1-mediated PI3K/AKT/mTOR, and cellular experiments using PI3K inhibitors such as LY294002 and pathway activity measurements or rescue experiments are needed to establish this mechanistic link. Current studies are limited by the small sample of human liver tissues, lack of REDD1 knockdown/KO model to contrast with overexpression, the inherent limitations of adenoviral vectors for cell-type-specific expression, generalizability beyond the BDL model. In future research, we will focus on validating these findings in more human liver sample size, and investigate the therapeutic effect of REDD1 overexpression and knockdown on more liver fibrosis models such as CCL4 models to explore the potential of REDD1-based therapies in clinical settings.

## 5 Conclusion

In summary, we identified that REDD1 alleviates liver fibrosis via the PI3K/AKT/mTOR pathway, and is a novel target for treating cholestatic liver fibrosis. Our study aims to provide a new perspective for the drug treatment of clinical cholestatic liver diseases.

## Data Availability

The datasets presented in this study can be found in online repositories. The names of the repository/repositories and accession number(s) can be found below: https://www.ncbi.nlm.nih.gov/, GSE218407.

## Supplementary material

Supplementary Figure S1 The location and abundance distribution of gene expression in control and BDL groups. **(A)** Reads align region map. Bottom of plot represent sample names, and left of plot represent the percentage of exon, intron, and intergenic. Most of the reads can be compared to the exon region. **(B)** EPKM distribution of all samples. The curves of six colors represent six samples, the abscissa indicates logarithm of FPKM of corresponding genes, and the ordinate indicates the probability density of genes. **(C)** Sample expression violin plot. Abscissa represents six samples, ordinate represents the logarithm of the gene expression FPKM.
