# Supplementary figures and images for: REDD1 attenuates cholestatic liver fibrosis and suppresses PI3K/AKT/mTOR pathway
Source: Front Med (Lausanne). 2025 Sep 24;12:1628260. doi: 10.3389/fmed.2025.1628260 (PMC12504318; doi:10.3389/fmed.2025.1628260)

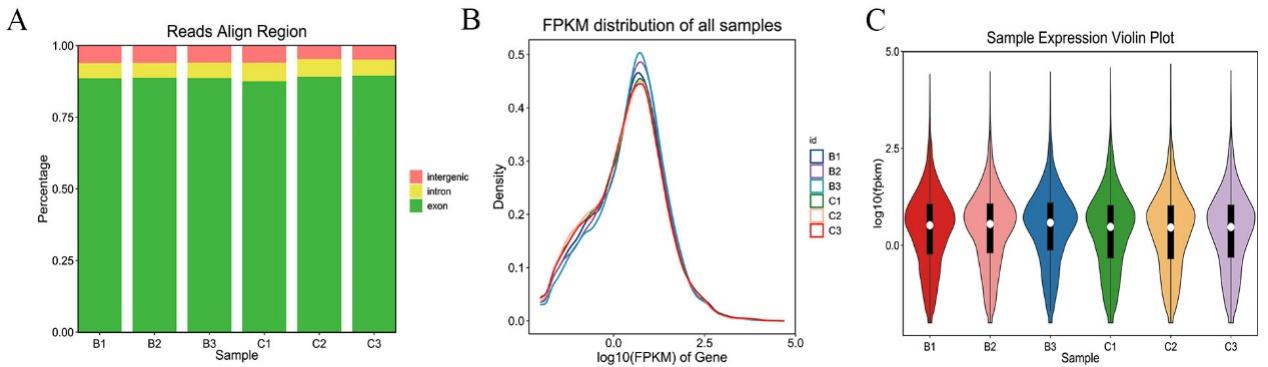

Supplement: Supplementary Figure S1 — The location and abundance distribution of gene expression in control and BDL groups. (A) Reads align region map. Bottom of plot represent sample names, and left of plot represent the percentage of exon, intron, and intergenic. Most of the reads can be compared to the exon region. (B) EPKM distribution of all samples. The curves of six colors represent six samples, the abscissa indicates logarithm of FPKM of corresponding genes, and the ordinate indicates the probability density of genes. (C) Sample expression violin plot. Abscissa represents six samples, ordinate represents the logarithm of the gene expression FPKM. [file Image_1.jpg]
